# Therapeutic comparison between treatments for Vulvar Lichen Sclerosus: study protocol of a randomized prospective and controlled trial

**DOI:** 10.1186/s12905-017-0414-y

**Published:** 2017-08-10

**Authors:** Renata A. Belotto, Maria Cristina Chavantes, João Paulo Tardivo, Roberto Euzébio dos Santos, Raquel Civolani Marques Fernandes, Anna Carolina Ratto Tempestini Horliana, Christiane Pavani, Daniela Fátima Teixeira da Silva

**Affiliations:** 1 Postgraduate Program in Biophotonics Applied to Health Sciences, Nove de Julho University/UNINOVE, 249 Vergueiro Street, Liberdade, São Paulo, SP 01504-001 Brazil; 2 Pérola Byington Hospital, 683 Brig. Luís Antônio Avenue, Bela Vista, São Paulo, SP 01318-000 Brazil; 3 Postgraduate Program in Medicine, Nove de Julho University/UNINOVE, 249 Vergueiro Street, Liberdade, São Paulo, SP 01504-001 Brazil; 4 ABC Medical School and Padre Anchieta Teaching Hospital, 470 Silva Jardim Street, Centre, São Bernardo do Campo, SP 09715-090 Brazil

**Keywords:** Collagen, Photobiomodulation, Photodynamic Therapy, Methylene blue, Corticosteroid

## Abstract

**Background:**

Vulvar lichen sclerosus (VLS) is a lymphocyte-mediated disease of unknown etiology that can cause intense itching as well stenosis, hindering the evacuation and urination. It can also limit the sex life due to severe local pruritus, pain and dyspareunia (pain during sexual intercourse). The standard treatment for this disease is the use of topical corticosteroids to reduce the clinical symptoms and to try to increase disease-free intervals. Photodynamic therapy (PDT), a treatment that associates a light radiation with a photosensitizing agent and photobiomodulation (PBM) are therapies that can promote effective immunomodulatory responses at the application site by means of photophysical and photochemical phenomena from the molecular to the systemic level, which promote their use in chronic dermatoses. The aim is to compare the effects of PDT, PBM, and topical corticosteroid in VLS evaluating clinical, histological, immunohistochemical and spectroscopic responses.

**Methods:**

The study is prospective, randomized and controlled, in a population of 60 women with histological diagnoses of VLS. There will be 3 treatments groups: PDT, PBM and topical corticosteroid (control group), where will be allocated by randomization 20 patients in each one. The clinical course will be monitored by measuring local temperature, itching, atrophy, and the area of the lesion. Histologically, the slides will be classified and will have the ordering of collagen fibers quantified. Immunohistochemical analysis will be done using the markers IFN-γ, TGF-β, CD4, CD8, IL-1, p53 and Ki-67. Finally, the spectroscopic evaluation will be done by reflectance. Descriptive and inferential statistical analyses will be conducted to compare the groups and make associations between different responses. The study is an open-label for patients with active symptomatic disease with a period of 1 year follow-up to determine the rate of recurrence in each groups.

**Discussion:**

The immunological effects of PDT and PBM are described by several authors in inflammatory skin diseases, stimulating the production and organization of the associated collagen. Thus, it is reasonable to determine the efficacy and safety of these new treatments in VLS, in comparison to the control group, analyzing the recurrence time, the impact on the optical properties of the skin, and the benefit to patients.

**Trial Registration:**

ClinicalTrials.gov: NCT02416531.

## Background

The pathogenesis of vulvar lichen sclerosus (VLS) is idiopathic, but some theories are described, such as the genetic theory, in which it is believed that about 22% of the lichen can be inherited [1]. There is the theory of hormonal changes due to decreased levels of dihydrotestosterone and androstenedione, and reduced activity of the enzyme 5 α reductase [2]. Autoimmune factors and oxidative stress have also been accepted due to the high association with autoimmune disease and the presence of highly specific antibodies against the extracellular matrix protein (ECM 1) [3]. Researchers have reported an autoimmune phenotype characterized by increased levels of Th1-specific cytokines, dense infiltration of T cells, and increased BIC/miR-155 expression [4]. Even though recent work has shown for the first time that vulvar lichen sclerosus is associated with 5-hydroxymethylation and altered expression of IDH enzymes, providing evidence for an epigenetic factor in the pathogenesis, literature is still reticent to establish it as an autoimmune disease [5]. Finally, there is the infectious theory of local factors, whose trauma and friction have been described, but as yet without evidence [6].

Vulvar lichen sclerosus affects the genital skin causing intense itching, whitening and atrophy, and this can cause stenosis, resulting in vulvodynia, pain on urination and bowel movements [7, 8]. It is the second leading cause of non-neoplastic vulvar disease, and is considered the most common cause of chronic vulvar disease with an estimated prevalence of 1:30–1:1000 [9]. The genital form is 5 to 10 times more frequent in women over 40 years and affected females outnumber males by 10:1 [10]. Anogenital involvement is 85% and extragenital is 15% [11]. The disease can affect children (1:900) and the clinical aspect is similar to that in adults, thus requiring long-term monitoring [11].

VLS has a malignant potential of around 4%, and is, therefore, considered as a means of vulvar carcinogenesis, with a recorded incidence of 32% to 76% of squamous cell carcinoma of the vulva adjacent to the lichen area [3, 7, 12, 13].

The diagnosis is clinical, but there may be uncertainty in the early stages of the disease and the main differential diagnoses are psoriasis, lichen planus, lichen simplex chronicus and benign mucous-membrane pemphigoid. A biopsy is recommended in cases that do not respond to treatment with topical corticosteroids, ulcerated lichen, or on suspicion of an invasive lesion [7, 14].

There is still no cure for VLS, so treatment is aimed at the symptoms, especially the vulvar itching, in order to prevent scarring and vulvar anatomical deformity, and to improve the quality of the sex life of patients, whom often report dyspareunia (painful intercourse), decreased orgasm and intercourse frequency compared to women who are not affected [9, 10]. Treatment is conservative using topical products [12], the gold-standard therapy is based on high-potency corticosteroid [15].

As the standard treatment can cause side effects, such as atrophy and permanent stretch marks, and still does not provide the cure, trying to find new therapies, per se*,* is an important challenging for the clinical practice. Effective alternatives that improve the excruciating skin process and optimize the quality of life in affected patients should be studied, and this is the motivation for this work, using photodynamic therapy (PDT) or photobiomodulation (PBM). There is no dosimetric protocol established for the treatment of VLS with PDT, nor with PBM. According to the literature, energy densities range from 9 to 150 J/cm^2^ and power densities from 40 to 700 mW/cm^2^ [16–21], without mentioning the studies that do not report the dosimetry used [22, 23].

There are few references in the literature about the action of PDT in vulvar lichen sclerosus [16–20, 22, 23], and till now there is no reference concerning PBM, although the anti-inflammatory and healing effects of PBM are documented in various medical and biological applications [24]. It is hoped that a prospective clinical study would contribute to more effective action in the control of this chronic skin disease, and possible understanding the mechanisms of action of these treatments.

It will be performed a comparative study among clobetasol propionate, photodynamic therapy, and photobiomodulation in patients with vulvar lichen sclerosus, using the following analyses:

- Clinical—by measuring the local temperature, pruritus, clamping and area of the lesion;
- Histological—by staining with hematoxylin & eosin;
- Immunohistochemical—using the markers IFN-γ, TGF-β, CD4, CD8, IL-1, p53, and Ki-67;
- Spectroscopic—by in vivo reflectance of skin.

## Methods/Design

The study is prospective, randomized, controlled, and conducted with 60 patients enrolled at Hospital Pérola Byington, São Paulo. The research will be undertaken in partnership with gynecologist Renata Ap. Belotto, CRM 59284.

After reading, understood and signed the Free and Informed Consent, the patients will undergo a biopsy for histological confirmation of VLS, samples for laboratory tests will be collected at the beginning and end of the study: complete blood count, fasting glucose, free T4, TSH, urea, creatinine, sodium, potassium and plasma cortisol.

### Inclusion criteria

The participants in this study will be only adult female (aged over 18 years), histological diagnosed with vulvar lichen sclerosus with a normal level of cortisol, confirmed by blood test.

### Exclusion criteria

Patients with any kind of ongoing cancer and/or AIDS or coagulopathy; pregnant or breastfeeding women; patients using corticosteroids, immunosuppressants or anticoagulants; patients with renal, hepatic or pulmonary–cardiovascular failure; patients who have undergone any kind of organ transplantation in the last three years.

### Randomization and dosimetry

A researcher not involved in the study will divide the patients into three groups by randomized order (Minitab 16, EUA). Then, opaque envelopes randomly containing information about application of photobiomodulation, photodynamic therapy or corticosteroid will be labelled with sequential numbers. The researcher responsible for treatments will open the first envelope and perform the procedure written therein. The internal contents will be revealed only after statistical analysis.

The dosimetry to be used in this study is based on a pilot clinical study performed by our group, which followed the recommendations from ASLMS [21]. In brief, the aim was to compare the effects of PBM and topical corticosteroid in VLS evaluating clinical response, itching, skin thickness (atrophy), and recurrence of disease after treatments. The study was prospective, randomized, and controlled in 20 women. CAAE number of Research Ethics Committee: 34,715,314.6.3001.0069. Corticosteroid group: clobetasol propionate 0.05% ointment applied once daily at a dose of 1 g/application for 4 weeks. PBM group: methylene blue 0.01% intralesional, laser λ = 660 nm, *P* = 100 mW, *I* = 510 mW/cm^2^, E = 4 J, exposure radiant = 20 J/cm^2^, *t* = 40 s, once a week for 4 weeks. The variation in intensity of itching was compared between groups, the corticosteroid group reduced the itching at 83.75%, and the PB group decreased 50.00%. The corticosteroid group showed significant variation in the skin thickness relative to PB (*p* = 0.006). This group had a reduction in thickness of the skin −27.51%, while in PB group showed an increase of 49.07%. The treatments were completed in December 2014 and until the month of September 2015 in the corticosteroid group there was 7 patients with no symptoms, while in the PB group there was 6 patients. In the corticosteroid group, the first recurrence was two months after the treatment, while the PBM was only 4 months later.

The groups to be studied and their dosimetry are showed at Table 1.

**Table 1:** Characteristics of the groups

Group	Number	Treatment	Parameters
GC	20	Corticosteroid over the whole vulva	Clobetasol propionate 0.05% ointment applied once daily at a dose of 1 g/application (1 g sachets) for 4 weeks
GPDT	20	Localized photodynamic therapy at 8 points of the vulva	Methylene blue 0.01% intralesional + lidocaine 2%, λ = 660 ± 10 nm, *P* = 100 mW, *I* = 510 mW/cm^2^, E = 4 J, RE = 20 J/cm^2^, *t* = 40 s, once a week for 4 weeks
GPBM	20	Localized photobiomodulation at 8 points of the vulva	The same parameters as for GPDT, except for the methylene blue, once a week for 4 weeks

Laser parameters: *λ* wavelength, *P* power, *I* irradiance, *E* energy, *RE* radiant exposure, *t* exposure time. Laser: Photon Laser III (DMC®, São Carlos/SP, Brazil). Methylene blue: Chimiolux 10 (DMC®, São Carlos/SP, Brazil). Clobetasol propionate: Drogaderma®, São Paulo/SP, Brazil)

**Fig. 1:**
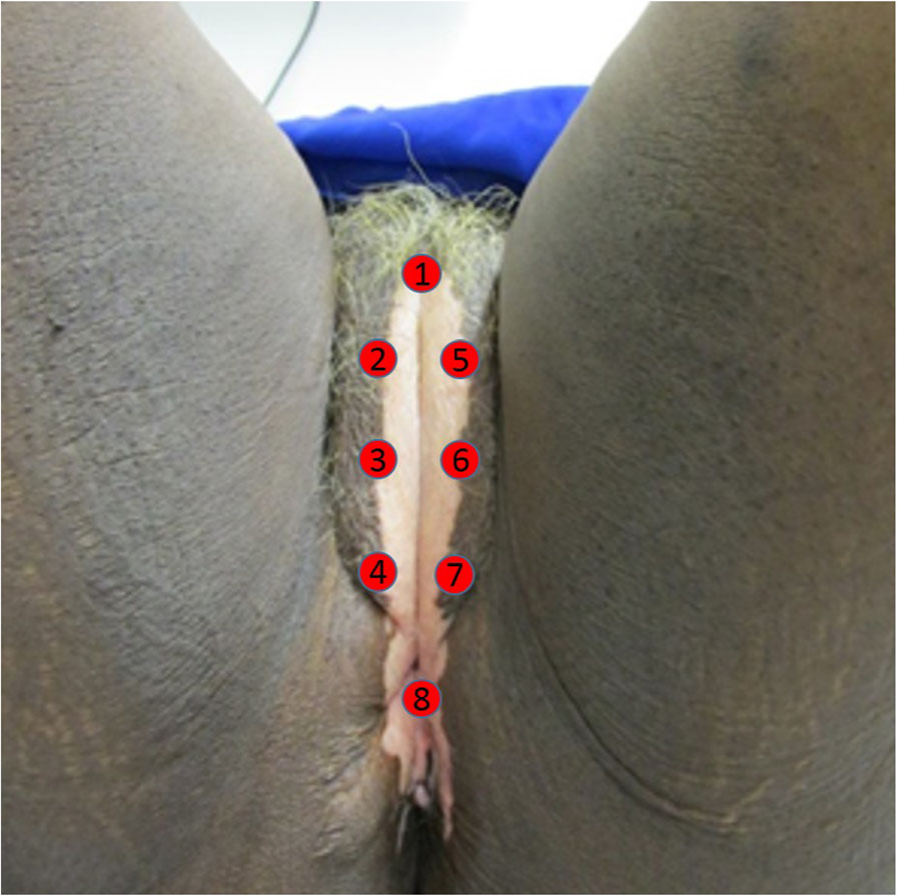
Photo of the vulva highlighting the 8 points of irradiation for PDT and PBM

The mode of application of photonic therapies will be by point, localized at 8 points of the vulva, as shown in Fig. 1.

Patients in the PDT group, upon arriving at the outpatient clinic, will be treated twice: first they will receive topical lidocaine gel 2% on the vulva and will return to the waiting room. Second: after 15 min, methylene blue with lidocaine will be injected, in a 1 to 1 ratio, at 8 points of vulva and so as to perform the PDT immediately. This procedure will be performed for the exact reason of minimizing the pain of the injection.

### Analyses

The control group (topical corticosteroid) will not be seen weekly because the standard treatment is performed by the patients themselves, in their own homes, for 30 days as recommended by the International Society for the Study of Vulvar Disease (ISSVD). In this way, all groups (GC, GPDT and GPBM) will receive treatment for 30 days and as biopsies before and after these 30 days will be compared. The study will be an open-label for patients with active symptomatic disease with a period of 12 months follow-up to determine the rate of recurrence in each arm.

#### Clinical

The temperature of the vulva will be measured with an infrared thermographic camera (C2, FLIR®, Nashua/NH, USA), which enables simultaneous measurement of the entire target area without physical contact. Measurements will be recorded as images in all sessions before, during, and after irradiation to observe the thermal fluctuation in the procedures.

In each session, the patients will be asked about the intensity of vulvar itching to assess its severity and duration, before and after irradiation, according to a visual analogical scale.

The vulvar skin clamping to evaluate the trophism will be done before irradiation at each session, using a digital caliper (Insize®, São Paulo/SP, Brazil), transversely and longitudinally in relation to the labia majora.

The area of the lesion will be monitored with a digital camera at every session (EOS Rebel T5, Canon®, Melville/NY, USA), before irradiation. To facilitate measurements, a metric scale will be placed on all vulvas for the photos. The areas of the lesions will be quantified using ImageJ software (National Institutes of Health, Maryland, USA).

The temperature, the intensity of itching, the clamping and the photographic images will be recorded for the patients of the GPDT and GPBM groups weekly for 30 days and for the GC group these measures will be done at first and 30th day.

If patients still report discomfort or undesirable symptoms from LEV after 30 days of treatment, they will continue with the same clinical protocol. If 60 days after the initiation of treatment they still remain symptomatic, the treatment that clinically appears to be better will be offered.

#### Histological

The biopsies will be performed at two points: at baseline to confirm the VLS and subsequent inclusion in the research protocol, and at the end of 30 days to investigate the prognosis after treatment. The skin fragments will be placed immediately in vials containing 4% buffered formalin, identified with the patient’s numbers and names, in accordance with the standard routine of the hospital, and sent to the Pathology Laboratory at the Hospital Pérola Byington. There will be no mention of the study group on the vials, so the analysis will be performed blind by one experienced pathologist.

Three sets of histological sections will be processed. One set will be for staining in hematoxylin and eosin in order to recognize cells and their components and to make inferences on the histology of the tissue under an ordinary optical microscope. The slides of each patient will be classified and the classifications will be assigned scores so that they can be statistically analyzed.

Another set will not be stained and will be deparaffinized for analysis of the ordering of collagen fibers under a polarized light microscope, according to an already established protocol [25]. The third and final set of histological sections will be used for the immunohistochemical technique, which is described in the next subsection. It is emphasized that the method used in the analysis of collagen ordering will be quantitative, by measuring the optical birefringence (Δn) of the samples, in nanometers.

#### Immunohistochemical

Once deparaffinized, the sections will be subjected to antigen retrieval, endogenous enzyme blocking, background blocking, incubations of antibodies, and counter-staining according to the instructions of the manufacturers of the IFN-γ, TGF-β, CD4, CD8, IL-1, p53, and Ki67 antibodies (Sigma-Aldrich®, St. Louis/MO, USA and Cell-Signaling®, Danvers/MA, USA). The cells that are positively stained by the immunohistochemical reaction will be counted by ImageJ software (National Institutes of Health, Maryland, USA) by two independent pathologists without prior knowledge of the experimental groups.

#### In-vivo reflectance spectroscopy

A portable spectrophotometer (400–900 nm) comprising a light source and a fiber-optic probe (USB2000, OceanOptics®, Dunedin/FL, USA) will be used directly on the surface of the vulvar skin in areas affected by VLS and in healthy areas of the same patients. Spectra will be taken in each session, just before the PDT and PBM. The patients in the corticosteroid group will have the spectrum recorded only twice—at baseline and after 30 days. Relative spectra will be obtained for the wavelengths corresponding to those of the therapeutic window, and the percentage of relative reflectance will be calculated.

### Statistical evaluation

Using the G* Power software version 3.1.9.2, the calculus of power analysis was determined by choosing F tests for repeated measures, within-between interaction, since 3 groups will be studied, and each group will be measure before and after treatments. The effect size was determined using the formula [26]:

$$ d=\frac{largest- smallest}{{\left(\frac{\sigma }{\sqrt{n}}\right)}^2} $$

The largest and smallest mean values, as well as the standard deviation were taken from the pilot clinical study [21]. The α error was set at 5% and the β error was set at 95%. The n value is the number of groups, i.e., three. According to the G* Power, a sample of 20 patients per group will be required for a power of 88.7%.

The profile of patients will be analyzed according to age, duration of disease, comorbidities and reported symptoms. A descriptive analysis will be conducted and presented by graph.

The clinical, histological, immunohistochemical, and spectroscopic findings will be submitted to inferential statistical analysis to verify the differences between the treatment groups. Associations between these results will also be analyzed. The significance chosen for the tests will be 95%.

First, the statistical distribution of the data will be evaluated. If the data conform to a Gaussian curve, parametric tests will be used. Graphs will be constructed in accordance with the means and standard deviations of the data.

However, if the data is unsuitable for a normal distribution, they will be analyzed with nonparametric tests. Box-type and quartile graphs, constructed according to the median of the results, will be used to present the data.

All data analysis will be performed using SPSS 22 Statistics software (IBM, USA).

### Global benefits for the patients

The criterion for recurrence will be pruritus, because it is the main patient complaint. The expected optical change in the skin is a reduction in reflection; lichen sclerosus leaves the skin depigmented, i.e. white, which is the color that most reflects. Therefore, as the symptoms diminish, we expect the skin to become less white, meaning that reflection also diminishes. We expect photonic therapies to decrease pruritus as much as the corticosteroid and, in addition, to not decrease the thickness of the skin, avoiding stenosis and pain.

## Discussion

The rare studies of PDT in intraepithelial neoplasia of the vulva have shown increased cytotoxic T lymphocyte in the treated area, as well as reduction in recurrence [27, 28]. The immunological effects of PDT and PBM are also described by several authors in inflammatory skin diseases, stimulating the production and organization of the associated collagen [24, 25, 29]. Thus, it is reasonable to determine the efficacy and safety of these new treatments in vulvar lichen sclerosus, analyzing the recurrence time, the impact on the optical properties of the skin, and the benefit to patients by clinical, histological, immunohistochemical and spectroscopic aspects.

Since high-potency topical corticosteroids are the first line treatment for VLS, clobetasol propionate 0.05% ointment is currently considered the gold standard. Approximately 60% of patients experience complete remission of symptoms, with consequent elimination of fissures, erosions, and hyperkeratosis [15]. Its use is effective, but the atrophy of the skin, scars, and hypopigmentation are irreversible with its use. Typically, a thin layer of steroid (finger tip unit) is applied once or twice daily for 2 to 4 weeks, then being reduced to three times a week, till withdrawal of the product [9].

PDT is a photochemical technique, and has been a treatment option in some gynecological and skin diseases [25, 29]. It consists of a combination of a photosensitizer and a light source, and these must be optically resonant. Its efficacy depends on the selectivity and retention of the photosensitizer, the intensity of the incident radiation, the transfer of excitation energy from the photosensitive agent and its oxidizing effect [30]. Specifically in VLS, its effectiveness has been varied, mainly due to the characteristics of the commonly used photosensitizers (methyl aminolevulinate and 5-aminolevulinic acid), which are topical, requiring periods ranging from two to five hours for sufficient production of porphyrin before the interaction with light [17, 21]. The lack of standardization of the various physical parameters of the radiation (light source, power, exposure time, energy and power densities) also contributes to the partial success of this treatment modality [31]. Adverse effects are reported, such as erythema, burning, and discomfort, even a few hours after therapy. Therefore, the association with intralesional anesthetic is common [17, 21, 31]. Nevertheless, the beneficial effects on the VLS are outstanding, such as the relief of symptoms for up to 6 months and/or the absence of the lesion [16–20].

The few studies of VLS treatments applying PDT are described with 5-aminolevulinic acid, a precursor of the photosensitive agent protoporphyrin IX, or its methyl ester, which is more lipophilic. However, at physiological pH, ALA forms zwitterions, which prevents its ability to cross biological barriers such as cell membranes, resulting in slow and non-homogeneous distribution in the target tissue, even as its high cost and phototoxicity [32].

On the other hand, methylene blue (MB) acting as the photosensitizer makes PDT more reasonable in clinical practice, especially in the Public Health System, since it has a high quantum yield of singlet oxygen and the ability to generate several radical species, it is highly photostable, easily eliminated from the body, presents minimal toxicity and low cost [33]. Moreover, the MB has an affinity for melanin, it is more lipophilic than ALA, actively binds to mitochondria and provides a mechanism for the reduction of the inflammatory response [34, 35]. The monomers and dimers of MB have distinct absorption spectra: the monomers have maximum absorption at 664 nm and the dimers at 590 nm. It is known that in a 20 μM aqueous solution there are only monomers present. A detailed review of the characteristics of MB can be found in the study of Tardivo et al. [36].

In PBM, the power density is not sufficient to induce biological activity dependent on the temperature increase, provided that the recommendations of the World Association for Laser Therapy (WALT) are followed [31]. It can be used with radiation sources that are coherent (lasers) or incoherent (lamps and LEDs). The basic biological mechanism that is currently accepted for explaining, although not completely, the effects observed in PBM is the absorption of red and infrared radiation by chromophores in mitochondria, in particular cytochrome c oxidase, and photoreceptors in the plasma membrane of cells, respectively [30]. The main reported effects are the reduction of pain and inflammation, optimization of tissue repair, tissue and nerve regeneration, and the repigmentation of skin lesions [24]. While these effects are desirable in the treatment of VLS, to date no work has been found associating it with PBM.

Nowadays we know that immunohistochemistry is an indispensable technique for solving differential diagnosis problems caused by basic routine staining of HE [32]. Although we know that the diagnosis of LEV is clinical, we insist to collect the biopsies so that we can cross-check the data (HE versus immunohistochemistry) for a better understanding of the disease and the mechanisms involved after the different therapies. We remember that VLS has a malignant potential of around 4%, and is, therefore, considered as a means of vulvar carcinogenesis, with a recorded incidence of 32% to 76% of squamous cell carcinoma of the vulva adjacent to the lichen area.

Studies performed by different groups has promoted a basis for associating antibodies for IFN-γ (interferon gamma), TGF-β (transforming growth factor beta), CD4, CD8, IL-1 (lymphocyte activating factor), p53, and Ki67 with vulvar lichen sclerosus [3, 5, 13, 33, 34]. The study of these cytokines and proteins will add to the understanding the biological mechanism triggered by photonic therapies.

Cytokine IFN-γ is the major interferon produced by lymphocytes stimulated by mitogens or antigens, and is related to immunoregulation. It is an inducer of IL-2 (interleukin 2), acting on the immune response profile of Th2 cells to Th1 cells, which control the homeostasis of the immune system. There are reports of increased IFN-γ staining in the VLS epidermis compared with the healthy vulva and non-vulvar skin, suggesting that this disease shares the characteristics of a chronic wound [33].

TGF-β protein plays a role in embryonic development, cell differentiation, hormone secretion, and immune function. Specifically during healing, it functions as a chemoattractant for neutrophils, macrophages and fibroblasts [35]. Although no difference has been found in the expression of TGF-β between lichen sclerosus and healthy skin, there are no studies comparing this marker as a function of different therapies, nor of its association with collagen synthesis in VLS [36].

The CD4–CD8 ratio is the ratio of T lymphocytes expressing CD4 antigens to those expressing CD8 antigens. This value is usually assessed in the diagnosis and stages of diseases that affect the immune system. The expression of these markers was pronounced in the dermal infiltrate of VLS [4].

Cytokine IL-1 is a soluble factor produced by monocytes, macrophages and other cells that activates T-lymphocytes and enhance their responses to mitogens and antigens. The biological effects of IL-1 include the ability to meet the requirements of macrophages necessary for activating T cells. The increased expression of this cytokine has also been reported in VLS [33].

Tumor-suppressant protein p53 is a nuclear phosphoprotein encoded by the p53 gene, and its normal function is to control cell proliferation and apoptosis. Ki-67 protein is present only in the active phase of the cell, making it a good marker for cell proliferation. Major expression of both markers was found in VLS, reflecting the risk of progression to malignancy [3, 13].

The demand for performing the challenging task of optically diagnosing and characterizing biological tissues can be attributed to the rapid development of laser applications in the field of medicine and, more recently, the use of LEDs (light-emitting diodes) [37]. Such applications require better understanding of the radiation–tissue interaction, and thus of the optical changes caused by the biological target in the light passing through it (photodiagnostics) and also the optical changes caused by the light radiation in the tissue (phototherapy).

To make inferences regarding the tissue structure based on measured optical characteristics is a complex task. However, the challenge is tempting and promising due to the fact that the optical characterization of biological tissue contains valuable diagnostic information, to the extent that there is a measurable difference between normal and pathological states, including preventively [38].

When the optical properties of the skin are altered, specifically the absorption and scattering coefficients, the intensity of reflectance is affected. The measurement of reflectance spectra has, therefore, been used to obtain the optical characteristics of the skin in vivo [39, 40]. Thus, it is possible to quantitatively evaluate the effect of different agents on skin condition by analysis of the spectral reflectance [41].

There are no reports in the literature on the reflectance of skin affected by VLS, which can contribute not only to the understanding of the mechanisms of the proposed phototherapies, but also to an improved optical knowledge of the tissue as a function of biological changes related to the stage of VLS [42]. Thus, since the spectroscopic study in VLS is unprecedented, it was decided to use reflectance spectroscopy because it is the easiest method in vivo. If significant differences are found between the spectra of different groups or in one patient depending on the duration of treatment, another study will be proposed for more detailed investigation to obtain the reduced absorption and scattering coefficients in the spectral range of the therapeutic window.

## Abbreviations

ECM 1: Extracellular matrix protein
ISSVD: International Society for the Study of Vulvar Disease
LEDs: Light-emitting diodes
MB: Methylene blue
PBM: Photobiomodulation
PDT: Photodynamic therapy
VLS: Vulvar lichen sclerosus
WALT: World Association for Laser Therapy
